# Adaptation of the mothers’ breastfeeding empowerment scale to Turkish society

**DOI:** 10.1017/S1463423626100863

**Published:** 2026-02-24

**Authors:** Zerrin Çiğdem, Leyla Kaya, Melike Yavaş Çelik

**Affiliations:** 1 Faculty of Health Sciences, İstanbul TopkapI Universitesi, Türkiye; 2 Faculty of Health Sciences, Health Sciences University, Türkiye; 3 Faculty of Health Sciences, Gaziantep University, Gaziantep, Türkiye

**Keywords:** breastfeeding, psychometric measurement, validity and reliability

## Abstract

**Aim:**

‘It was aimed to establish the validity and reliability of the “Mothers’ Breastfeeding Empowerment Scale”’.

**Method:**

Content validity was evaluated with the content validity index (CVI) agreed upon by experts. Exploratory factor analysis (EFA) and confirmatory factor analysis (CFA) were used for construct validity. Before factor analysis, the sample size was decided using KMO and Bartlett’s Sphericity test. For the reliability study of the scale, Cronbach’s Alpha coefficient and total score correlation coefficients of parallel forms were examined.

**Results:**

While 49.9% of the infants were between 4–6 months old, the average age of the infants was determined as 3.64 ± 1.77. 70.6% of the mothers were between the ages of 26–43 and the average age of the mothers was 28.83 ± 5.38. In the factor analysis it was found that item loadings of the scale was between 0.57 and 0.95. Also, the fit values of the scale were within acceptable limits. Additionally, it was determined that there was a high-level positive relationship between the scale used for the parallel form and our scale.

**Conclusion:**

The use of the scale in Turkish society is valid and reliable. Validity and reliability analyses support this.

## Introduction

Breastfeeding is very important in the entire growth and development process (Wang *et al*., 2021). Breastfeeding is also the optimal method to reduce morbidity and mortality in children (Agampodi *et al*., 2021). The World Health Organization (WHO) recommends that breastfeeding should start within the first hour after birth, that infants should be only breastfed until they are 6 months old, and that infants should be breastfed for at least 2 years (World Health Organization, 2021). In the last decade, the prevalence of only breastfeeding for infants in the first 6 months has increased by 10%, reaching 48% worldwide (World Health Organization, 2023). The WHO has targeted breastfeeding rates to be 70% by 2030. To achieve this breastfeeding goal, the barriers women and their families face to breastfeeding must be eliminated (World Health Organization, 2023). To increase breastfeeding rates, interventions that can empower mothers and encourage effective breastfeeding are needed (Sinha *et al*., 2015). Successful breastfeeding is influenced by several psychological factors, including mothers’ attention to breastfeeding, breastfeeding education for mothers, perceived support for breastfeeding, breastfeeding self-efficacy, and breastfeeding empowerment. Breastfeeding self-efficacy and breastfeeding empowerment are psychological and motivational factors that influence the continuity and success of breastfeeding (Mohammadi *et al*., 2022). Therefore, it is very important and necessary to use a valid and reliable tool to strengthen breastfeeding. Many scales have been developed and used in this direction (Tokat *et al*., 2010; Ekşioğlu and Çeber, 2011). It is also a very important parameter for breastfeeding success in evaluating breastfeeding problems and adequacy. There are scales developed in this direction and are used today (Dolgun *et al*., 2018). These tools developed to evaluate breastfeeding do not evaluate many parameters together, such as adequate breastfeeding knowledge and skills, perceived breastfeeding adequacy, belief in breastfeeding, ability to overcome breastfeeding problems, efforts to receive family support, and breastfeeding self-efficacy (Dolgun *et al*., 2018; Mohammadi *et al*., 2022). Moreover, these tools are quite limited. The challenges mothers face with breastfeeding have not been addressed in detail. Furthermore, there are a significant number of women of childbearing age in our country who need breastfeeding support. Given that these women marry young and that first-time mothers are less likely to breastfeed, having tools that address this issue would make the work of healthcare professionals easier. The low breastfeeding rates in our country also highlight the need for a tool on this topic. (TUIK, 2023; Alan, 2025). In this study, it was aimed to establish the validity and reliability of the ‘Mothers’ Breastfeeding Empowerment Scale’.

## Method

### Type of study

The research was conducted methodologically. The reliability and validity of the Breastfeeding Empowerment Scale (Mohammadi *et al*., 2022) developed by Mohammadi *et al*. was tested.

### Determination of the population and sample

The population consisted of all mothers who applied to a hospital. The sample consisted of mothers who had infants between 1 and 6 months of age, who did not have any problems that would prevent them from participating in the study, who were breastfeeding, and who volunteered to participate in the study (*n* = 377). The number of samples was determined by calculating the number of items in the scale (37 × 10 = 370). However, the study was completed by reaching 377 people. Additionally, Kaiser-Meyer-Olkin (KMO) = 0.942, Chi-square = 5989.701, *p* = 0.001 values gave very strong results. The information in the literature supports determining the sample in this way (Ercan and Kan, 2004; Vefikuluçay Yılmaz and Terzioğlu, 2011; Seçer, 2015). Power calculations were performed using the study by Mohammadi *et al*. (2022). The sample size for the power analysis was calculated using the G*Power programme. The first error type was 0.05, the Cohen’s effect size was 0.3, and the sample size was 242. Based on these inputs, the calculated power was found to be 95%. But, taking into account data losses and other analyses, this study was completed with 377 people.

### Collection of research data

Research data was collected with the following forms and scales.

**Question form:** This form includes demographic data of the mother and infant and questions about breastfeeding.

### It was tested scale for validity and reliability

**Mothers’ Breastfeeding Empowerment Scale (MBES)**: The scale was developed by Mohammadi *et al.* (Mohammadi *et al.*, 2022) There are no reverse-coded questions in the scale items. The scale can be applied to mothers who have infants aged between 1 and 6 months and who are breastfeeding. The scale is evaluated in Likert-type scale. The scale is scored as: strongly disagree (1), disagree (2), undecided (3), agree (4), strongly agree (5). The scale has 37 items in total. The scale consists of 6 sub-dimensions: Adequate breastfeeding knowledge and skill (1–11 items), perceived breastfeeding adequacy (12–15 items), informed belief in breastfeeding value (16–22 items), overcoming breastfeeding problems (23–29 items), negotiation for receiving family support (30–34 items), and breastfeeding self‑efficacy (35–37 items). As the score increases, breastfeeding power increases. (Mohammadi *et al*., 2022). Cronbach’s alpha values for the scale and its sub-dimensions were found as follows: Adequate breastfeeding knowledge and skill: 0.88, perceived breastfeeding adequacy: 0.78, informed belief in breastfeeding value: 0.77, overcoming breastfeeding problems: 0.77, negotiation for receiving family support: 0.77, breastfeeding self‑efficacy: 0.77, whole scale: 0.93.

### Scale used as parallel from

**Breastfeeding Self-Efficacy Scale (BSES):** It is a scale consisting of 33 items developed by Dennis to measure breastfeeding self-efficacy. It includes the determination of mothers’ breastfeeding skills and their beliefs and behaviours regarding breastfeeding. The validity and reliability study of the Turkish form of the scale was conducted by Ekşioğlu and Çeber (2011). All items are preceded by the phrase ‘I can always’ and anchored with a 5-point Likert-type scale where 1 indicates not at all confident and 5 indicates always confident. As recommended by Bandura (1977), all items are presented positively, and scores are summed to produce a range from 33 to 165, with higher scores indicating higher levels of breastfeeding self-efficacy. Content validity of the BSES was based on the literature, interviews with breastfeeding mothers, and expert judgement using a method recommended. After a pilot test, an initial psychometric assessment was conducted with a convenience sample of 130 Canadian breastfeeding women who completed questionnaires in-hospital and at 6 weeks postpartum (Dennis and Faux, 1999). Cronbach’s alpha coefficient for the scale was 0.96, with 73% of all corrected item-total correlations ranging between 0.30 and 0.70. Responses were subjected to principal components analysis with a varimax rotation, yielding the theorized technique and intrapersonal subscales. Technique is defined as the physical action a mother performs and represents certain tasks necessary for successful breastfeeding. Intrapersonal thoughts are defined as a mother’s perceptions of breastfeeding and include attitudes and beliefs related to a successful breastfeeding experience. Support for predictive validity was demonstrated through significant differences in BSES scores and infant feeding patterns at 6 weeks postpartum. In the current study, the BSES was completed at 1, 4, and 8 weeks postpartum (Dennis and Faux, 1999; Ekşioğlu and Çeber, 2011).

## Analysing data

The data obtained within the scope of the study were transferred to the computer environment, and Statistical Package for Social Sciences (SPSS) 25.0 and LISREL 8.80 package programmes were used for statistical analysis. In descriptive statistics, number, percentage, mean, and standard deviation values were used. Content validity was evaluated with the content validity index (CVI), which was agreed upon by experts. For construct validity, EFA, CFA, and CFA fit indices were examined. Before factor analysis, the sample size was decided using KMO and Bartlett’s Sphericity test. For the reliability study of the scale, Cronbach’s alpha coefficient and Pearson correlation coefficients were examined.

## Application of research

First, permission was obtained from the researchers who developed MBES. Later, the scale was translated into Turkish. Then, a pilot study was conducted to check the face validity of the translated scale, and it was evaluated whether the items of the scale were understood. Additionally, 10 experts were consulted to evaluate the scale. These experts evaluated whether the scale was suitable for Turkish society and whether it was necessary for breastfeeding success. Experts stated that the scale was appropriate and that it would be useful to conduct this study. Then, data was collected to analyse the scale between 01 August 2023 and 05 October 2023. After data was collected, the construct validity of the scale was evaluated with factor analysis and reliability analyses were performed.

## Ethics of research

Ethics committee approval was obtained from the Ethics Committee of a University. Written and verbal consent was obtained from the mothers. The research was conducted in accordance with the Principles of the Declaration of Helsinki.

## Results

### Participants

While 49.9% of the infants were between 4–6 months old, the average age of the infants was determined as 3.64 ± 1.77. While 70.6% of the mothers participating in the study were between the ages of 26–43, the average age of the mothers was 28.83 ± 5.38. It was found that of the mothers, 100% were married, 76.1% were housewives, 95.5% had a moderate economic status, 31.6% were university graduates or had postgraduate education, and 59.7% had 1 or 2 pregnancies (Table 1).

**Table 1.** Characteristics of mothers and infants and questions regarding breastfeeding

Data		*n* = 377	% = 100
**Age mean: 28.83 ± 5.38**	18–25	111	29.4
	26–43	266	70.6
**Marital status**	Yes	377	100
	No	0	0
**Job**	Housewife	287	76.1
	Civil servant	51	13.5
	Private sector employee	39	10.4
**Economic situation of the family**	Income over expense	7	1.9
	Income and expenditure balanced	360	95.5
	Revenue less than expense	10	2.7
**Educational status**	Literate and primary school graduate	45	11.9
	Secondary school graduate	105	27.9
	High school graduate	108	28.6
	University and postgraduate graduate	119	31.6
**Number of pregnancies**	1–2 times	226	59.7
	3–4 times	119	31.6
	5–7 times	32	8.5
**Age of infant mean: 3.64 ± 1.77**	1–1.5 month	68	18.0
	2–3.5 month	121	32.1
	4–6 month	188	49.9

## Validity

### Language adaptation

The suitability of the translation was determined as appropriate by expert opinion. Additionally, the statements sent to the owner of the scale were approved by the owner of the scale (Table 2).

**Table 2.** Validity and reliability analyses of MBES

Analyses made	Results
**Validity analysis**	
**Language adaptation**	A translation-back translation technique was used for language adaptation. The scale items were translated from English to Turkish by two experts who knew the English language and the cultural structure of the two communities well. Then, the translations made from Turkish to English by two different experts in their fields were compared, and appropriate sentences were arranged.
**Face validity (** ***n*** **= 10)**	The comprehensibility of the items in the scale was tested by 10 Turkish students studying abroad. Students were first asked to evaluate MBAS in Turkish. They were asked to evaluate the MBES in English 18 days after the initial assessment. Between the two evaluations (first evaluation: 168.46 ± 13.84, second evaluation: 168.37 ± 15.20), it was seen that there was no statistically significant result between the average scores given by the students to the scale questions and their answers were close to each other (*p* > 0.05).
**Content validity**	It is evaluated using Kendall’s W test. As a result of this test, it was determined that the opinions of the experts did not change between each other, they were in agreement, and there was no statistically significant relationship between the opinions (*p* > 0.05).
**Construct validity (** ***n*** **= 377)**	**Exploratory factor analysis (EFA)** Kaiser-Meyer-Olkin (KMO) = 0.942, X2 = 5989.701, *p* = 0.001 **Confirmatory factor analysis (CFA)** X2/df: 225.20/614=0.3, *p* = 0.01 Goodness of fit index (GFI) = 0.92 Adjusted goodness of fit index (AGFI) = 0.88 Comparative fit index (CFI) = 0.96 Normed fit index (NFI) = 0.95 Non-normed fit index (NNFI) = 0.99 Incremental fit index(IFI) = 0.99 Relative fit index = 0.99 Standardized root mean square residual (SRMR) = 0.04 Root mean square error of approximation(RMSEA) = 0.01
**Reliability analysis**	
**Item total correlation**	The correlation between the sub-dimensions of the scale was evaluated as strong. Sub-dimensions 1 and MBES *r* = 0.93, *p* = 0.01, sub-dimensions 2 and MBES *r* = 0.70, *p* = 0.01, sub-dimensions 3 and MBES *r* = 0.77, *p* = 0.01, sub-dimensions 4 and MBES *r* = 0.80, *p* = 0.01, sub-dimensions 5 and MBES *r* = 0.83, *p* = 0.01, sub-dimensions 6 and MBES *r* = 0.79, *p* = 0.01.
**Cronbach’s alpha**	Cronbach’s alpha values for the scale and its sub-dimensions were found as follows: Adequate breastfeeding knowledge and skill: 0.88, perceived breastfeeding adequacy: 0.78, informed belief in breastfeeding value: 0.77, overcoming breastfeeding problems: 0.77, negotiation for receiving family support: 0.77, breastfeeding self‑efficacy: 0.77, whole scale: 0.93.
**Parallel form reliability**	In the analysis conducted for parallel form reliability, a very strong positive relationship was found between BSES and MBES (*r* = 0.72, *p* = 0.01).

## Face validity

The comprehensibility of the items in the scale was tested by 10 Turkish students studying abroad. Students were first asked to evaluate MBAS in Turkish. They were asked to evaluate the MBES in English 18 days after the initial assessment. Between the two evaluations (first evaluation: 168.46 ± 13.84, second evaluation: 168.37 ± 15.20), it was seen that there was no statistically significant result between the average scores given by the students to the scale questions and their answers were close to each other (*p* > 0.05) (Table 2).

## Content validity

As a result of this test, it was determined that the experts’ opinions did not change from each other, they agreed, and there was no statistically significant relationship between the opinions (*p* > 0.05). Experts rated the scale items (1 point) between much change needed and (4 points) as very appropriate. It was determined that the average score given to the scale items by the experts was between 3.81 ± 0.60 and 4.00 ± 0.00 points. CVI of 0.95 was determined, demonstrating very good content validity (Table 2).

## Construct validity

Exploratory factor analysis (EFA): Kaiser-Meyer-Olkin (KMO) = 0.942, Chi-square = 5989.701, *p* = 0.001 values gave very strong results. It was decided that other analyses could be started based on these values (Table 2).

**Figure 1.**
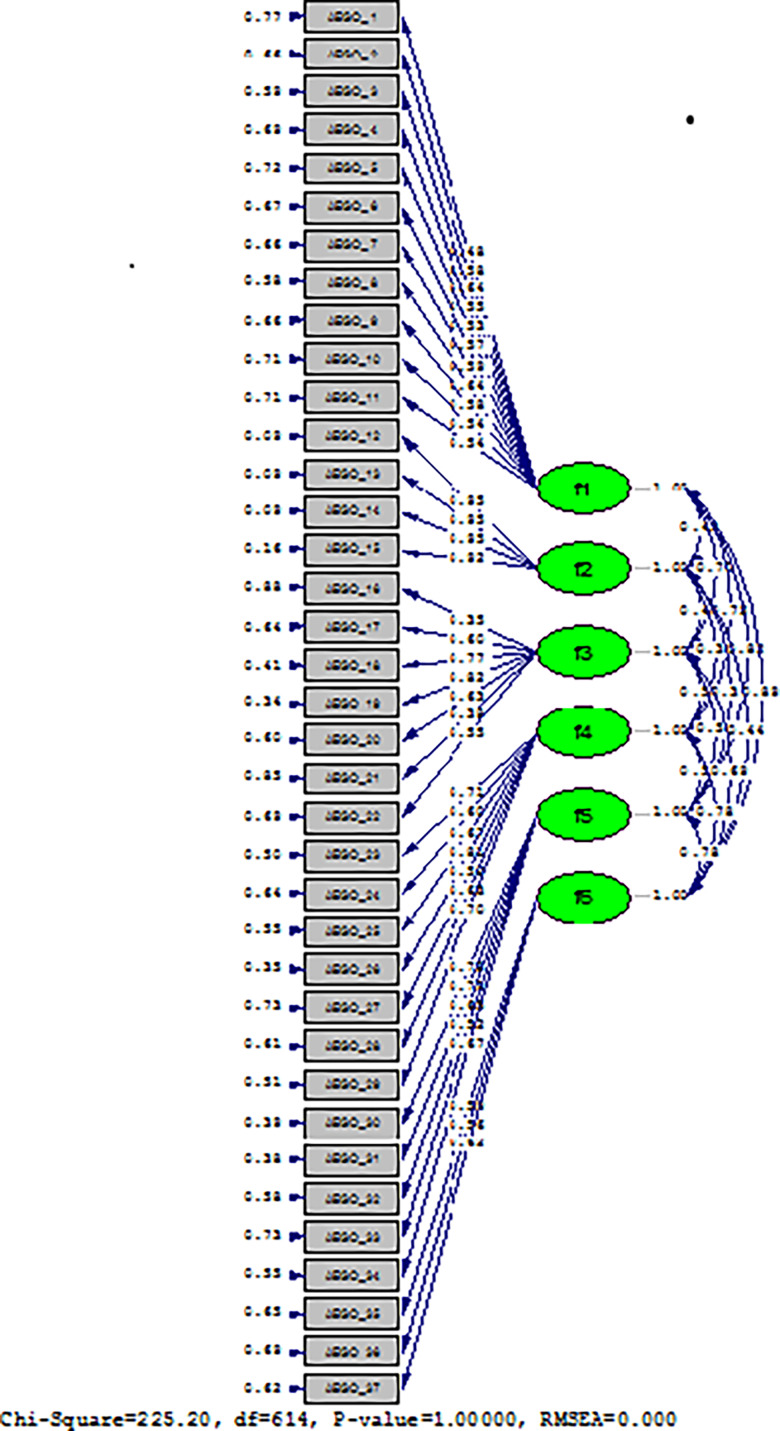
Factor analysis of the scale.

Confirmatory factor analysis (CFA): The fit index values made on the LISREL programme are as follows: X2/df:225.20/614 = 0.3, *p* = 0.001. Goodness of fit index (GFI) = 0.92, adjusted goodness of fit index (AGFI) = 0.88, comparative fit index (CFI) = 0.96, normed fit index (NFI) = 0.95, fit index (NNFI) = 0.99, incremental fit index (IFI) = 0.99, relative fit = 0.99, standardized root mean square residual (SRMR) = 0.04, root mean square error of approximation (RMSEA) = 0.01 (Table 2). In the factor analysis, we conducted for construct validity, the item loadings of the MBES were found to be at acceptable values between 0.35 and 0.82. In addition, all items and subscales in the scale were preserved. No items were removed (Table 3) (Figure1).

**Table 3.** Item loadings of MBES

Items	Sub-dimensions					
	Adequate breastfeeding knowledge and skill	Perceived breastfeeding adequacy	Overcoming breastfeeding problems	Informed belief in breastfeeding	Negotiation for receiving family support	Breastfeeding self‑efficacy
Item 1	0.48					
Item 2	0.58					
Item 3	0.64					
Item 4	0.55					
Item 5	0.53					
Item 6	0.57					
Item 7	0.59					
Item 8	0.64					
Item 9	0.58					
Item 10	0.54					
Item 11	0.54					
Item 12		0.95				
Item 13		0.95				
Item 14		0.95				
Item 15		0.92				
Item 16			0.35			
Item 17			0.60			
Item 18			0.77			
Item 19			0.82			
Item 20			0.63			
Item 21			0.38			
Item 22			0.55			
Item 23				0.71		
Item 24				0.60		
Item 25				0.67		
Item 26				0.81		
Item 27				0.52		
Item 28				0.63		
Item 29				0.70		
Item 30					0.78	
Item 31					0.79	
Item 32					0.65	
Item 33					0.52	
Item 34					0.67	
Item 35						0.59
Item 36						0.56
Item 37						0.62

## Reliability

### Item total correlation

The correlation between the sub-dimensions of the scale was evaluated as strong. Sub-dimensions 1 and MBES *r* = 0.93, *p* = 0.01, sub-dimensions 2 and MBES *r* = 0.70, *p* = 0.01, sub-dimensions 3 and MBES *r* = 0.77, *p* = 0.01, sub-dimensions 4 and MBES *r* = 0.80, *p* = 0.01, sub-dimensions 5 and MBES *r* = 0.83, *p* = 0.01, and sub-dimensions 6 and MBES *r* = 0.79, *p* = 0.01 (Table 2).

### Cronbach’s alpha

Cronbach’s alpha values for the scale and its sub-dimensions were found as follows:

Adequate breastfeeding knowledge and skill: 0.88, perceived breastfeeding adequacy: 0.78, informed belief in breastfeeding value: 0.77, overcoming breastfeeding problems: 0.77, negotiation for receiving family support: 0.77, breastfeeding self‑efficacy: 0.77, and total scale: 0.93 (Table 2).

### Parallel forms

In the analysis conducted for parallel form reliability, a strong positive relationship was found between BSES and MBES (*r* = 72, *p* = 0.01) (Table 2). The relationship between the sub-dimensions of MBES and BSES was quite strong. The correlation values between the sub-dimensions of MBES and BSES were as follows: Sub-dimensions 1 (Adequate breastfeeding knowledge and skill) BSES *r* = 0.69, *p* = 0.01, sub-dimensions 2 (Perceived breastfeeding adequacy) and BSES *r* = 0.52, *p* = 0.01, sub-dimensions 3 (Informed belief in breastfeeding value) and BSES *r* = 0.54, *p* = 0.01, sub-dimensions 4 (Overcoming breastfeeding problems) and BSES *r* = 0.56, *p* = 0.01, sub-dimensions 5 (Negotiation for receiving family support) and BSES *r* = 0.58, *p* = 0.01, and sub-dimensions 6 (Breastfeeding self‑efficacy) and BSES *r* = 0.59, *p* = 0.01 (Table 4).

**Table 4.** Correlation between MBES and BSES

Scales	* ***r***, ***p***	Adequate breastfeeding knowledge and skill	Perceived breastfeeding adequacy	Overcoming breastfeeding problems	Informed belief in breastfeeding	Negotiation for receiving family support	Breastfeeding self‑efficacy	Total
**BSES**	*r*	0.69	0.52	0.54	0.56	0.59	0.58	0.72
	*p*	0.01	0.01	0.01	0.01	0.01	0.01	0.01

*Correlation is significant at the 0.01 level (2-tailed).

## Discussion

In this study we conducted to test the validity and reliability of MBES, we determined that the scale is a compatible tool suitable for Turkish society. With construct validity, it was determined that all items of the scale worked and factor loadings were within valid limits. In addition, the fact that our scale has a completely positive relationship with the parallel form scale has proven to us that our scale is a reliable tool. Cronbach’s alpha coefficient, which is another reliability analysis, was analysed as reliable values in the scale sub-dimensions and the overall total of the scale. According to all these analyses, we can say that our scale is a valid and reliable tool. Similar results were obtained in the study conducted by Mohammadi *et al*., who developed the scale (Mohammadi *et al*., 2022).

In our country, there are many tool such as breastfeeding self-efficacy scale (Dennis and Faux, 1999; Ekşioğlu and Çeber, 2011), LATCH breastfeeding assessment tool (Jensen *et al*., 1994; Yenal and Okumuş, 2003), Bristol Breastfeeding Evaluation Scale (Ingram *et al*., 2015; Dolgun *et al*., 2018). However, all these scales evaluate knowledge, attitudes, and behaviours regarding breastfeeding separately or some parameters together. However, there is no tool that evaluates breastfeeding behaviour, self-efficacy, knowledge, and attitude together. However, MBES offers the opportunity to evaluate adequate breastfeeding knowledge and skills, perceived breastfeeding adequacy, informed belief in breastfeeding value, overcoming breastfeeding problems, negotiation for receiving family support, and breastfeeding self-efficacy altogether. For this reason, instead of evaluating using several tools, using a single scale can help us save labour and time. Therefore, it would be beneficial to popularize and promote the use of this scale.

In addition to reliability and validity analyses, scores from the scale were also analysed. The participants’ mother’s MBES score was found to be quite high. Accordingly, we can say that mothers are strong in breastfeeding. In fact, one of the factors that led to this result may be that the majority of mothers had a university or undergraduate education. This study supported Bu şüphelerimizi başka bir analizle desteklemiş bulunmaktayız. When we compared the total average scores of mothers from MBES in terms of their educational status, we found that mothers with university degrees and postgraduate education received higher scores than mothers with secondary school degrees, resulting in a statistically significant result. The role of education level, which is one of the demographic characteristics, is very important in continuing breastfeeding (Dennis 2002; McLeod *et al*., 2002). It has been determined that mothers with higher education levels are more successful in continuing breastfeeding (Dennis 2002; Blyth *et al*., 2002). Additionally, studies have shown that mothers with lower education levels terminate breastfeeding much earlier (Ong *et al*., 2001; Wagner *et al*., 2006). In their study, Küçükoğlu and Çelebioğlu (2014) determined that university graduates had higher breastfeeding success. In this case, it would be correct to say that education level is a factor that affects mothers’ breastfeeding success. Then we can say that it is also necessary to increase the education level of women in order to strengthen breastfeeding. Another finding of ours is that the average MBES total score of mothers working in the private sector is statistically significantly higher than that of civil servant mothers. This result may be due to the fact that mothers working in the private sector have their own workplaces, or it may be due to the fact that the majority of mothers working in the private sector quit their jobs and focused entirely on infant care.

## Conclusion

The 37-item MBES is valid and reliable. Midwives, nurses, and lactation consultants can use MBES to measure breastfeeding and improve needs-based.

## References

[ref1] Agampodi TC , Dharmasoma NK , Koralagedara IS , Dissanayaka T , Warnasekara J , Agampodi SB and Perez-Escamilla R (2021) Barriers for early initiation and exclusive breastfeeding up to six months in predominantly rural Sri Lanka: a need to strengthen policy implementation. International Breastfeeding Journal 16(1), 1–12. 33832496 10.1186/s13006-021-00378-0 PMC8034146

[ref2] Alan S (2025) TÜİK Verileri Işığında Aile Yapısındaki Değişimlerin Sosyolojik Değerlendirmesi. Afyon Kocatepe Üniversitesi Sosyal Bilimler Dergisi 27(Aile Özel Sayısı), 177–198.

[ref3] Blyth R , Creedy DK , Dennis CL , Moyle W , Pratt J and De Vries SM (2002) Effect of maternal confidence on breastfeeding duration: an application of breastfeeding selfefficacy theory. Birth 29, 278–284. 12484390 10.1046/j.1523-536x.2002.00202.x

[ref5] Dennis CL (2002) Breastfeeding initiation and duration: a 1990-2000 literature review. Journal of Obstetric, Gynecologic, and Neonatal Nursing 31, 12–32. 10.1111/j.1552-6909.2002.tb00019.x 11843016

[ref4] Dennis CL and Faux S (1999) Development and psychometric testing of the breastfeeding self-efficacy scale. Research in Nursing & Health 22(5), 399–409. 10520192 10.1002/(sici)1098-240x(199910)22:5<399::aid-nur6>3.0.co;2-4

[ref6] Dolgun G , İnal S , Erdim L and Korkut S (2018) Reliability and validity of the Bristol Breastfeeding Assessment Tool in the Turkish population. Midwifery 57, 47–53 10.1016/j.midw.2017.10.007 29197786

[ref7] Ekşioğlu AB and Çeber E (2011) Translation and validation of the breastfeeding self-efficacy scale into Turkish. Midwifery 27(6), 246–253 10.1016/j.midw.2010.10.009 21145148

[ref8] Ercan İ and Kan İ (2004) Ölçeklerde Güvenilirlik ve Geçerlilik. Uludağ Üniversitesi Tıp Fakültesi Dergisi 30, 211–216.

[ref10] Ingram J , Johnson D , Copeland M , Churchill C and Taylor H (2015) The development of a new breastfeeding assessment tool and the relationship with breastfeeding selfefficacy. Midwifery 31, 132–137. 10.1016/j.midw.2014.07.001 25061006 PMC4275601

[ref9] Jensen D , Wallace S and Kelsay P (1994) LATCH: breastfeeding charting system and documentation tool. JOGNN 23(1), 27–32. 8176525 10.1111/j.1552-6909.1994.tb01847.x

[ref11] Kavle JA , Picolo M , Buccini G , Barros I , Dillaway CH and Pérez-Escamilla R (2019) Strengthening counseling on barriers to exclusive breastfeeding through use of job aids in Nampula, Mozambique. PLoS One 14(12), e0224939. 31790430 10.1371/journal.pone.0224939 PMC6886792

[ref12] Küçükoğlu S and Çelebioğlu A (2014). HASTA YENĠDOĞANLARIN ANNELERİNİN EMZİRME ÖZ YETERLİLİK DÜZEYİ VE EMZİRME BAŞARILARININ İNCELENMESİ Erciyes Üniversitesi Sağlık Bilimleri Fakültesi Dergisi 2(1), 1–11

[ref13] McLeod D , Pullon S and Cookson M (2002) Factors influencing continuation of breastfeeding in a cohort of women. Journal of Human Lactation 1, 335–343. 10.1177/089033402237906 12449049

[ref14] Mohammadi F , Kohan S and Heidari Z (2022) Development and psychometric evaluation of the Mothers’ breastfeeding empowerment scale: a mixed methods study. Nursing and Midwifery Studies 11(4), 240.

[ref15] Ong G , Yap M , Li FL and Choo TB (2001) Impact of working status on breastfeeding in Singapore: evidence from the national breastfeeding survey. European Journal of Public Health 2005 15, 424–430 10.1093/eurpub/cki030 16030134

[ref16] Seçer İ (2015) SPSS VE LISREL ile Pratik Veri Analizi. Ankara: Anı Yayıncılık, pp. 211–258.

[ref17] Sinha B , Chowdhury R , Sankar M. J , Martines J , Taneja S , Mazumder S , Rollins N , Bahl R and Bhandari N (2015). Interventions to improve breastfeeding outcomes: a systematic review and meta-analysis. Acta Paediatrica 104, 114–134. 10.1111/apa.13127 26183031

[ref18] Tokat MA , Okumuş H and Dennis CL (2010) Translation and psychometric assessment of the Breast-feeding self-efficacy scale—short form among pregnant and postnatal women in Turkey. Midwifery 26(1), 101–108 18541350 10.1016/j.midw.2008.04.002

[ref19] (TUIK) Turkish Statistical Institute (2023). Available at https://data.tuik.gov.tr/Bulten/Index?p=Turkiye-Saglik-Arastirmasi-2022-49747 (accessed 10 March 2025).

[ref20] Vefikuluçay Yılmaz D and Terzioğlu F (2011). Development and psychometric evaluation of ageism attitude scale among the university students. Turkish Journal of Geriatrics/Türk Geriatri Dergisi 14(3), 259–268.

[ref21] Wagner CL , Wagner MT , Ebeling M , Chatman KG , Cohen M and Hulsey TC (2006) The role of personality and other factors in a mother’s decision to initiate breastfeeding. Journal of Human Lactation 22, 16–21 16467284 10.1177/0890334405283624

[ref26] Wang Y , Zhao T , Zhang Y , Li S and Cong X (2021) Positive effects of kangaroo mother care on long-term breastfeeding rates, growth, and neurodevelopment in preterm infants. Breastfeeding medicine 16(4), 282–291 33533688 10.1089/bfm.2020.0358

[ref22] World Health Organization (2021) Breastfeeding. Available at https://www.who.int/health-topics/breastfeeding#tab=tab_1 (accessed 24 October 2023).

[ref23] World Health Organization (2023) Joint Statement by UNICEF Executive Director and WHO Director-General on the Occasion of World Breastfeeding Week. Available at https://www.who.int/news/item/01-08-2023-joint-statement-by-unicef-executive-director-catherine-russell-and-who-director-general-dr-tedros-adhanom-ghebreyesus-on-the-occasion-of-world-breastfeeding-week (accessed 24 October 2023).

[ref24] Wu Y , Wang Y , Hu J , Dang Y , Zhang Y , Qi X , Tian Q , Wang A and Li, Y (2021). Breastfeeding competency scale (BCS); development and validation of an evaluation instrument on breastfeeding competency in third trimester pregnancy. BMC Pregnancy and Childbirth 21, 1–11. 33663421 10.1186/s12884-021-03664-1 PMC7934416

[ref25] Yenal K and Okumuş H (2003) LATCH Emzirme tanılama ölçeğinin güvenirliğini inceleyen bir çalışma. HEMAR-G Dergisi 5(1), 38–44.

